# Can the pan-immune-inflammation value predict gram negative bloodstream infection-related 30-day mortality in solid organ transplant patients?

**DOI:** 10.1186/s12879-024-09413-x

**Published:** 2024-05-24

**Authors:** Çağlayan Merve Ayaz, Özge Turhan, Vural Taner Yılmaz, Haydar Adanır, Beyza Sezer, Dilara Öğünç

**Affiliations:** 1 Department of Infectious Diseases and Clinical Microbiology, Faculty of Medicine, Akdeniz University, Antalya, Turkey; 2 Department of Internal Medicine, Division of Nephrology, Faculty of Medicine, Akdeniz University, Antalya, Turkey; 3 Department of Internal Medicine, Division of Gastroenterology, Faculty of Medicine, Akdeniz University, Antalya, Turkey; 4 Department of Medical Microbiology, Faculty of Medicine, Akdeniz University, Antalya, Turkey

**Keywords:** Solid organ transplantation, Gram negative bacterial infection, Multidrug resistance, Pan-immune-inflammation value, Mortality

## Abstract

**Background:**

The recently used pan-immune-inflammation value (PIV) has not been adequately studied as a predictive marker for mortality in immunosuppressed patients. The aim of this study was to evaluate the usefulness of baseline PIV level as a predictor of 30-day mortality in solid organ transplant (SOT) recipients with gram negative bloodstream infections (GN-BSI).

**Methods:**

This retrospective, cross-sectional study was conducted between January 1, 2019, and December 31, 2022, in 1104 SOT recipients. During the study period, 118 GN-BSI were recorded in 113 patients. Clinical, epidemiological, and laboratory data were collected, and mortality rates (30-day and all-cause) were recorded.

**Results:**

The 113 recipients had a median age of 50 years [interquartile range (IQR) 37.5–61.5 years] with a male predominance (*n* = 72, 63.7%). The three most common microorganisms were as follows: 46 isolates (38.9%) of *Escherichia coli*, 41 (34.7%) of *Klebsiella pneumoniae*, and 12 (10.2%) of *Acinetobacter baumannii*. In 44.9% and 35.6% of the isolates, production of *extended-spectrum beta-lactamases* and carbapenem resistance were detected, respectively. The incidence of carbapenem-resistant GN-BSI was higher in liver recipients than in renal recipients (*n* = 27, 69.2% vs *n* = 13, 17.6%, *p* < 0.001). All-cause and 30-day mortality rates after GN-BSI were 26.5% (*n* = 30), and 16.8% (*n* = 19), respectively. In the group with GN-BSI-related 30-day mortality, the median PIV level was significantly lower (327.3, IQR 64.8–795.4 vs. 1049.6, IQR 338.6–2177.1; *p* = 0.002). The binary logistic regression analysis identified low PIV level [hazard ratio (HR) = 0.93, 95% confidence interval (CI) 0.86–0.99; *p* = 0.04], and increased age (HR = 1.05, 95% CI 1.01–1.09; *p* = 0.002) as factors associated with 30-day mortality. The receiver operating characteristic analysis revealed that PIV could determine the GN-BSI-related 30-day mortality with area under curve (AUC): 0.723, 95% CI 0.597–0.848, *p* = 0.0005.

**Conclusions:**

PIV is a simple and inexpensive biomarker that can be used to estimate mortality in immunosuppressed patients, but the results need to be interpreted carefully.

## Background

Solid organ transplantation is one of the best lifesaving ways to increase survival in individuals with organ failure [1]. Immunosuppressive drugs used for grafts survival bring with them an unintended increased risk of infection, as a result of deterioration of immune system functions [2]. Therefore, the management of infections, especially gram negative (GN) bloodstream infections (BSI), which are the main causes of morbidity and mortality in solid organ transplant (SOT) patients, is crucial [3]. In previous studies, recipient age, comorbidities, source of infection, inappropriate antibiotic use, intensive care admission, history of previous surgery, re-transplantation, low platelet count, high white blood cell count, creatinine level, and aspartate aminotransferase level, polymicrobial infections, mycophenolate mofetil (MMF) use were related to mortality in SOT patients with BSI [4].

Inflammation is an essential immune response that plays a vital role in preventing microbial infections and other foreign invaders from harming the host [5]. Currently, a large number of biomarkers that can be easily measured in peripheral blood are used to assess the indirect effect of inflammation, prognosis, and treatment response in diseases. The pan-immune-inflammation value (PIV), which is mostly and recently used scoring system that includes neutrophil, monocyte, lymphocyte, and platelet counts in clinical trials in cancer, rheumatological diseases, and other inflammatory processes; has not been adequately studied as a predictive marker for worse outcomes and prognosis in immunocompromised patients [6–10]. Prior studies have generally found that elevated PIV levels are an indicator of worse scenario [6, 8–11]. There are few studies using PIV in infectious diseases, probably because of the difficulty of interpreting the result due to the many factors affecting blood parameters. A recent study examined the association between PIV and other prognostic markers and mortality in intensive care patients hospitalized with sepsis. The results demonstrated that PIV was not associated with mortality, but rather caused a decrease in survival time [12]. Various biomarkers, including the neutrophil to lymphocyte ratio (NLR) and the systemic inflammation response index, have been used as diagnostic biomarkers for BSI [13]. Another study used NLR as a predictor for the severity of GN-BSI [14]. In a study investigating deaths related to coronavirus disease 2019 (COVID-19), NLR, PIV, systemic immune-inflammation index, and absolute eosinopenia were used as biomarkers [6].

Solid organ transplant patients are an example of individuals with secondary immunodeficiencies [15]. To the best of our knowledge, no previous studies have investigated the relationship between PIV and mortality and prognosis in SOT patients with BSI. This cost-effective test, which can be easily calculated from a complete blood count, may have the potential benefit to predict the severity and prognosis of the infection, thereby possibly modifying the approach and management of the SOT patients. In this study, we aim to identify risk factors and evaluate the utility of the relatively new parameter PIV in clinical practice as a 30-day mortality biomarker in GN-BSI.

## Methods

### Study design and patients

This retrospective, cross-sectional study was conducted at Akdeniz University Hospital in Turkey between January 1, 2019, and December 31, 2022, in 1104 SOT (874 renal, 213 liver, and 17 heart) recipients. The analysis of prognostic factors only considered the first episode of GN-BSI from each patient that occurred during the observational period. All patients included in the study were over 18 years old. The PIV was calculated based on the blood parameters obtained on the day of the first positive blood culture. Study protocol was approved (No: 70904504/205) and the need for written informed consent was waived by the Clinical Research Ethics Committee of Akdeniz University due to retrospective nature of the study. The research was conducted in accordance with the Declarations of Helsinki.

### Data collection

The medical records of patients were used to collect baseline clinical, epidemiological, and laboratory data. The following factors were considered: gender, age, comorbidities (including diabetes mellitus, hypertension, cardiovascular diseases, chronic kidney disease, chronic liver disease, chronic pulmonary diseases, rheumatologic diseases, malignancy, and cerebrovascular accident), graft donation from deceased or living donors, type and date of transplantation, number and type of immunosuppressive treatment, date and location of GN-BSI diagnosis, duration of hospitalization before GN-BSI, site of primary infection, presence of other concomitant BSI, microbiological characteristics, and laboratory data including PIV. Mortality rates (30-day and all-cause) were also recorded. This study did not include treatment selection and efficacy for GN-BSI as they were beyond its scope.

### Definitions

The Centers for Disease Control and Prevention criteria were used to determine the presence of BSI [16]. Early-onset BSI is defined as an infection that occurs within the first 30 days of SOT or less. Late-onset infection is defined as an infection that occurs after this period. When a positive bacterium is detected in combination with a confirmed GN-BSI, the term "presence of other concomitant BSI" is added to this infection [17]. The PIV was calculated using the following formula: [neutrophil count (10^3^/mmc) × platelet count (10^3^/mmc) × monocyte count (10^3^/mmc)] divided by the lymphocyte count (10^3^/mmc) [7].

### Statistical analysis

All categorical data are presented as numbers (percentages) and all numerical data as medians (interquartile range, IQR). Categorical variables were compared using Pearson’s χ2 test or Fisher’s exact test, while non-normally distributed continuous numerical variables were compared using the Mann–Whitney U test, where appropriate. Bonferroni correction was made if necessary, in univariate analysis. A binary logistic regression analysis was conducted to assess the correlation between variables and 30-day mortality related to GN-BSI. To provide better clarity on the confidence interval (CI), divide the PIV by 100. Binary logistic regression was used to evaluate the Hazard ratio (HR) with 95% CI for the impact of demographic, clinical, and laboratory predictors on 30-day mortality. To avoid overfitting the modelling, a regression model was designed that includes age, gender, and PIV, considering the outcome investigated in the study. Statistical significance was determined at a value of *p* < 0.05. The capacity of PIV to predict GN-BSI-related 30-day mortality was analyzed using receiver operating characteristics (ROC) curve analysis. The study reported sensitivity, specificity, positive and negative predictive values (NPV, PPV) when a significant cut-off value was present, with a 95% CI and a 5% significance level (*p* < 0.05). The statistical analysis was performed using SPSS version 24.0 (IBM SPSS Statistics, IBM Corporation, Armonk, NY, United States).

## Results

A total of 1104 patients were screened between 1 January 2019 and 31 December 2022. During the study period, 118 GN-BSI were recorded in 113 SOT patients. The 113 recipients had a median age of 50 years (IQR 37.5–61.5 years) and a male predominance (*n* = 72, 63.7%; Table 1).

**Table 1:** The characteristics of study population

Characteristics	All patients (n, %) 113 (100)	Survivors (n, %) 94 (83.2)	Deceased (n, %) 19 (16.8)	*p*
Median age (years, IQR)	50.0 (37.5–61.5)	47.0 (35.5–61.3)	58.0 (52.0–62.0)	**0.002**
Male gender (n, %)	72 (63.7)	61 (64.9)	11 (57.9)	0.60
The type of transplant (n, %)				** < 0.001**
Liver	39 (34.5)	25 (26.6)	14 (73.7)	
Kidney	74 (65.5)	69 (73.4)	5 (26.3)	
The type of donor (n, %)				**0.003**
Living	90 (79.6)	80 (85.1)	10 (52.6)	
Deceased	23 (20.4)	14 (14.9)	9 (47.4)	
Number of comorbidities (n, %)				
2	70 (61.9)	59 (62.8)	11 (57.9)	0.70
≥ 3	26 (23.0)	23 (24.5)	3 (15.8)	0.60
Number of IS drugs (n, %)				
1	13 (11.5)	7 (7.4)	6 (31.6)	**0.008**
2	26 (23.0)	20 (21.3)	6 (31.6)	0.37
3	71 (62.8)	66 (70.2)	5 (26.3)	** < 0.001**
Type of IS drugs (n, %)				
Steroids	105 (92.9)	88 (93.6)	17 (89.5)	0.62
Mycophenolate mofetil	75 (66.4)	69 (73.4)	6 (31.6)	** < 0.001**
Tacrolimus	89 (78.8)	80 (85.1)	9 (47.4)	**0.001**
Cyclosporin	8 (7.1)	5 (5.3)	3 (15.8)	0.13
Everolimus	6 (5.3)	5 (5.3)	1 (5.3)	1.0
Sepsis/septic shock (n, %)	23 (20.4)	4 (4.3)	19 (82.6)	** < 0.001**
Neutrophil, median (IQR)	8.37 (4.32–14.11)	9.22 (5.20–14.83)	5.52 (2.80–10.91)	**0.02**
Lymphocyte, median (IQR)	0.45 (0.24–0.77)	0.51(0.26–0.78)	0.25 (0.12–0.70)	**0.03**
Monocyte, median (IQR)	0.40 (0.20–0.68)	0.45 (0.21–0.71)	0.21 (0.11–0.43)	**0.01**
Platelet, median (IQR)	136.0 (76.5–208.5)	152.0 (95.0–216.0)	63.0 (40.0–127.0)	** < 0.001**
Median CRP mg/L (IQR)	137.3 (88–181.6)	133.2 (87.3–180.3)	138.0 (92.0–206.9)	0.26
Median PIV (IQR)	928.7 (277.6–1900.0)	1049.6 (338.6–2177.1)	327.3 (64.8–795.4)	**0.002**

*Abbreviations: IQR* Interquartile range, *IS* Immunosuppressive, *CRP* C reactive protein, *PIV* Pan-immune-inflammation. Bold indicates *p* < 0.05

Among the 113 SOT recipients, 74 (65.5%) had kidney transplant, and 29 (34.5%) had liver transplant. All of the patients had at least one comorbidity, and 90 transplants (79.6%) were from living donors. At the time of GN-BSI onset, 0.9% (*n* = 1) of the patients was taking four immunosuppressive drugs, 62.8% (*n* = 71) were taking three, %23 (*n* = 26) were taking two, and 11.5% (*n* = 13) were taking one drug. Immunosuppressive treatment was changed (reduced in all but 1 patient) in 21.2% (*n* = 24) of patients and no significant difference in mortality was found in those patients (*p* = 0.23). In 9.7% (*n* = 11) of patients, immunosuppressive treatment was reduced after the diagnosis of GN-BSI. The patients on three immunosuppressive drugs had a significantly higher PIV value than those on one or two drugs (*p* = 0.001; medians were 3.38, 3.58, and 11.84, respectively). Table 1 presents the demographic, clinical, and laboratory characteristics of the patients, as well as the results of the univariate analysis of factors associated with GN-BSI-related 30-day mortality.

All variables included in PIV were found to be lower in deceased patients than in survivors. In the group with GN-BSI-related 30-day mortality, the median PIV level was significantly lower (327.3, IQR 64.8–795.4 vs. 1049.6, IQR 338.6–2177.1; *p* = 0.002) (Table 1). All-cause and 30-day mortality rates after GN-BSI were 26.5% (*n* = 30), and 16.8% (*n* = 19), respectively. The mortality rate was 18.4% (*n* = 9) for patients with early-onset bacteremia, 35.9% (*n* = 14) for liver transplant recipients and 26.3% (*n* = 5) for kidney transplant recipients.

The GN-BSI was detected in 41.6% (*n* = 47) of the patients who were admitted to the outpatient clinics. A total of 17 (11.5%) patients with hospital-acquired GN-BSI were followed up in the intensive care unit. The diagnosis of GN-BSI after transplantation typically occurs 39 days (IQR 11.0–152.5 days) after the procedure, while the median time to diagnosis of GN-BSI in hospitalized patients was 11 days (IQR 7.0–25.0 days). The three most common microorganisms were as follows: 46 isolates (38.9%) of *Escherichia coli*, 41 (34.7%) of *Klebsiella pneumoniae*, and 12 of (10.2%) *Acinetobacter baumannii*. The predominant primary site of infections in the first episode of GN-BSI were urinary tract (54.0%) and surgical site (31.9%). While there is less mortality in bacteremia originating from the urinary system, there is a higher mortality rate in surgical site and respiratory tract infections (*p* < 0.001). If a source of infection was detected, necessary procedures were performed in all patients to resolve it. *Extended-spectrum beta-lactamases* (*ESBLs*) and carbapenem resistance were noted in 44.9% and 35.6% of the isolates, respectively. The incidence of carbapenem-resistant (CR) GN-BSI was higher in liver recipients than in renal recipients (*n* = 27, 69.2% vs *n* = 13, 17.6%, *p* < 0.001). Early-onset bacteremia was occurred in 49 (43.4%) patients. Table 2 shows the microbiological characteristics of the microorganisms.

**Table 2:** The microbiological characteristics of the microorganisms

**Isolated bacteria (n, %)**			***p***
*Escherichia coli*	46 (39.0)		
*Klebsiella pneumoniae*	41 (34.7)		
*Acinetobacter baumannii*	12 (10.2)		
*Pseudomonas aeruginosa*	6 (5.1)		
*Enterobacter spp*	6 (5.1)		
*Klebsiella spp*	4 (3.4)		
Others* (Proteus mirabilis* + *Morganella morganii)*	3 (2.5)		
**Monomicrobial infection (n, %)**	108 (95.6)		0.20
**Source of infection (n, %)**	**Survivors**	**Deceased**	** < 0.001**
Urinary tract	60 (63.8)^a^	1 (5.3)^b^	
Surgical site	23 (24.5)^a^	13 (68.4)^b^	
Respiratory tract	5 (5.3)^a^	5 (26.3)^b^	
Intraabdominal/biliary	4 (4.3)^a^	- ^a^	
Others (Catheter related + unknown)	2 (2.2)^a^	- ^a^	
Hospital-acquired infection (n, %)	48 (72.7)	18 (27.3)	** < 0.001**
*ESBLs* ( +) bacilli, number of cases (%)	50 (53.2)	2 (10.5)	**0.001**
CR bacilli, number of cases (%)	24 (25.5)	16 (84.2)	** < 0.001**
Early-onset bacteremia (n, %)	40 (42.6)	9 (47.4)	0.70

*Abbreviations:* *ESBLs* Extended-spectrum beta-lactamases, *CR* Carbapenem resistant. a and b letter denotes a subset of 30-day mortality categories whose column proportions differ statistically significant from each other. Bold indicates *p* < 0.05

**Fig. 1:**
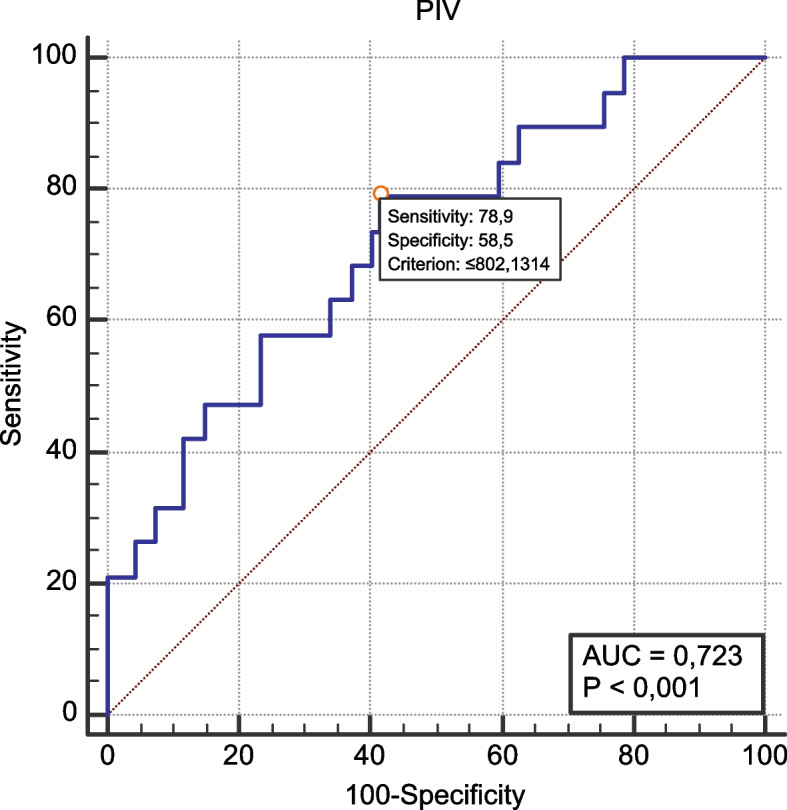
The ROC curve of PIV to predict GN-BSI-related 30-day mortality in SOT patients. Abbreviations: PIV: Pan-immune-inflammation; AUC: Area under curve

The ROC curve of PIV to predict GN-BSI-related 30-day mortality in SOT patients is shown in Fig. 1. The threshold of PIV was set at 802.1. The area under the curve was 0.723 (95% CI 0.597–0.848, *p* = 0.0005), with a sensitivity and specificity for predicting mortality of 78.9% and 58.5%, respectively. The results demonstrated a PPV of 27.8% and a NPV of 93.2%.

Binary logistic regression analysis (Table 3) revealed that low PIV level (HR = 0.93, 95% CI 0.86–0.99; *p* = 0.04) and increased age (HR = 1.05, 95% CI 1.01–1.09; *p* = 0.002) were significant predictors of 30-day mortality in GN-BSI among SOT patients.

**Table 3:** Factors affecting gram-negative bloodstream infection-related 30-day mortality

Variables	*p*	HR 95% CI
Age (per year)	**0.02**	1.05 (1.01–1.09)
Male gender	0.82	0.88 (0.30–2.65)
PIV	**0.04**	0.93 (0.86–0.99)

*Abbreviations: PIV* Pan-immune-inflammation. Regression modelling was performed using age, gender, and PIV as variables. Bold indicates *p* < 0.05

## Discussion

There is an increased risk of infections caused by GN-BSI in SOT patients [18]. For appropriate antibiotic selection and patient management, it is crucial to understand infection dynamics and predictors associated with mortality. The present study demonstrated that mortality due to GN-BSI was significantly higher in SOT patients with increasing recipient age and low PIV levels.

The PIV is often used to evaluate the association between tumor-mediated inflammation-immune response and survival [7, 11]. Studies have investigated the relationship between PIV and various inflammatory conditions, including long-term survival after myocardial infarction, frailty, and diagnosis and prognosis in rheumatological diseases [6, 8–10]. Previous studies reported that patients with high PIV levels measured at the onset of the condition considered indexed have a significantly increased likelihood of poor prognosis, treatment non-response and poor outcomes [11]. Contrary to other studies, in this study, it was observed that higher PIV level at the time of GN-BSI onset in SOT patients was associated with better prognosis. The adverse effects of immunosuppressive drugs (e.g. neutropenia with MMF, T-cell depletion with anti-thymocyte globulin; leukopenia, leukocytosis, and thrombocytopenia with tacrolimus; lymphopenia with steroids and MMF), and antimicrobial prophylaxis (e.g. neutropenia or thrombocytopenia with valganciclovir or ganciclovir; pancytopenia, or thrombocytopenia with trimethoprim-sulfamethoxazole) have been identified as possible causes of this finding. Furthermore, inflammatory changes caused by bacterial infection itself (e.g. leukocytosis, neutropenia, lymphopenia, and thrombocytopenia) may also contribute. The severity of the illness, age-related changes in the immune system, organ dysfunction and the patient's nutritional status are other factors that may influence this.

In a study that evaluated the prognosis of COVID-19 patients, no significant relationship was found between PIV and the risk of developing severe COVID-19 [6]. In another study involving 82 sepsis patients, high PIV was found to be associated with decreased survival time, along with other prognostic markers such as Sequential Organ Failure Assessment (SOFA), Acute Physiology and Chronic Health Evaluation II (APACHE II), procalcitonin, and lactate elevations [12]. Although there are no comparable head-to-head studies to evaluate the predictive ability of PIV in the immunosuppressed patient group with infection, it shows promise for evaluating mortality. When utilized for this specific purpose, it is of paramount importance to exercise caution when interpreting the result, as blood parameters may be influenced by a multitude of external factors.

The distribution of infections seen in SOT patients is proportionate to the anatomic position of the transplanted organ. While urinary tract infection is more commonly found in renal transplant patients, surgical site and intra-abdominal infections are reported in liver transplant patients [19]. *Escherichia coli* and *K. pneumoniae* were the predominant GN bacteria among these SOT population with BSI. Although the spectrum of bacteria varies between centers, similar results were found with other studies [19–22]. The prevalence of CR-GN bacteria in the study cohort is consistent with previous reports in deceased and liver transplanted patients [18, 21, 22].

Immunosuppressive therapy is one of the cornerstones of organ transplantation. Developments in this area have significantly improved patient and graft survival [23]. In the present study, we found a negative correlation between the degree of immunosuppression and the risk of 30-day mortality, similar to a recent report [20]. However, this result was not interpreted as a decrease in mortality with increasing degree of immunosuppression. It was concluded that patients whose immunosuppressive treatment was not reduced may have milder infections, better general condition, and fewer side effects. The management of immunosuppressive drugs in the presence of an active infection varies between centers [24–26]. In our center, this decision is usually based on the general condition of the patient, laboratory findings, the severity of the infection and the condition of the transplanted organ. For example, tacrolimus and MMF treatments were discontinued in patients with poor general condition and severe disease.

The study has several limitations, including its retrospective nature, being conducted in a single center, and the presence of confounding factors that affect blood parameters. Furthermore, the sample size is relatively small, and the number of deceased patients is limited. Consequently, the mortality analysis were carried out with a limited number of variables to limit the width of the confidence intervals. In addition, as the study was retrospective did not include specific prognostic scores (SOFA score, Pitt bacteremia score or others…) and immune markers such as enzymes, immune cell subtypes, ILs, TNF-alpha, CDs, we could not compare PIV with other specific markers. Furthermore, the limitations of the retrospective data precluded a comparison of the prognostic impact of PIV with other prognostic scores (e.g. the SOFA score or the Pitt bacteremia score…) or immune markers, including enzymes, immune cell subtypes, ILs, TNF-alpha, and CDs. Therefore, well-designed prospective studies with larger sample sizes are needed, especially to understand the relationship between other prognostic factors and GN-BSI outcomes. However, PIV can be used as an affordable, easy, and minimally invasive biomarker to assess unfavorable outcomes in SOT recipients.

## Conclusions

Pan-immune-inflammation value, an accessible and inexpensive biomarker, shows potential to predict mortality in immunosuppressed patients, but the result should be interpreted with caution. However, further prospective research is needed to explore the promise of PIV as a prognostic marker in SOT recipients.

## Data Availability

The datasets used and/or analyzed during the current study is available from the corresponding author on reasonable request.

## Abbreviations

APACHE II: Acute Physiology and Chronic Health Evaluation II
BSI: Bloodstream infections
CI: Confidence interval
COVID-19: Coronavirus disease 2019
ESBLs: *Extended-spectrum beta-lactamases*
GN: Gram negative
HR: Hazard ratio
IQR: Interquartile range
MMF: Mycophenolate mofetil
NLR: Neutrophil to lymphocyte ratio
NPV: Negative predictive values
PIV: Pan-immune-inflammation value
PPV: Positive predictive values
ROC: Receiver operating characteristics
SOFA: Sequential Organ Failure Assessment
SOT: Solid organ transplant
